# Hypertension management algorithm for type 2 diabetic patients applied in primary care

**DOI:** 10.1186/1758-5996-5-52

**Published:** 2013-09-12

**Authors:** Luciana V Viana, Cristiane B Leitão, Maria F Grillo, Ennio P C C Rocha, Juliana K Brenner, Rogerio Friedman, Jorge L Gross

**Affiliations:** 1 Endocrine Division and Primary Care Unit of Hospital de Clinicas de Porto Alegre and Federal University of Rio Grande do Sul, Porto Alegre 90035-003, RS, Brazil

**Keywords:** Type 2 diabetes, Hypertension, JNC 7, ADA guidelines, ‘Real life’

## Abstract

**Background:**

Hypertension frequently coexists with type 2 diabetes (DM), and increases the risk of cardiovascular outcomes. The aim of the study was to obtain/maintain blood pressure (BP) goals (ADA/JNC 7) according to a stepwise algorithm using the medication supplied by the Brazilian government.

**Methods:**

A one-year, single-arm interventional study conducted with type 2 diabetes patients. Intervention consisted of intensification of lifestyle changes and sequential prescription of drugs: diuretic; ACE inhibitors; β-adrenergic blocking agent and calcium channel blocking agent if BP >130/80 mmHg.

**Results:**

Seventy-eight patients completed the trial. During intervention, the number of antihypertensive tablets rose (3.6 ± 3.5 vs. 5.9 ± 3.5 pills/patient; p <0.001), as the number of antihypertensive classes increased (1.8 ± 1.0 vs. 2.70 ± 1.2; p < 0.01) and the overall drop of BP was 11 mmHg for SBP (145.0 ± 22.8 vs. 133.7 ± 20.9 mmHg; p < 0.01) and 5 mmHg for DBP (78.7 ± 11.5 vs. 73.7 ± 10.5 mmHg; p = 0.001). Although the number of patients with BP in target almost doubled [14 (18.7%) vs. 30 (38.5%) p = 0.008], less than 40% of the patients achieved the proposed goals.

**Conclusions:**

A BP algorithm applied to type 2 diabetic and hypertensive patients is able to lower BP, however more than half of the patients did not achieve the ADA/JNC 7 targets demonstrating the complexity of BP control in this population.

**Trial registration:**

ClinicalTrials.gov: NCT06260

## Introduction

Type 2 diabetes and hypertension frequently coexist, and patients with this combination are at a higher risk for cardiovascular events [1]. United Kingdom Prospective Diabetes Study (UKPDS) concluded that tight blood pressure (BP) control in patients with type 2 diabetes and hypertension is able to reduce micro and macrovascular diabetic complications [2]. However, strict BP control in this population, as advised by The Seventh Report of the Joint National Committee on Prevention, Detection, Evaluation and Treatment of High Blood Pressure (JNC 7) and American Diabetes Association (ADA), with a target BP lower than 130/80 mmHg is difficult to obtain, therefore multiple medications are often required [3-5].

In Brazil, hypertension was present in 81% of the patients with diabetes participating in the HiperDia System - a program developed to provide antihypertensive and antidiabetic medication in primary care units throughout the country [6]. The aim of this study was to analyze if it is possible to obtain and maintain BP goals (ADA and JNC 7) with an aggressive BP lowering strategy, according to a stepwise algorithm for BP using the medication supplied by the Brazilian government (HiperDia System).

### Patients and methods

Consecutive adult patients (greater than 18 years of age) with type 2 diabetes, who were regularly attending a primary care unit (at least 2 consultation in the last during the 6-month period before the screening visit), were invited to participate in the study. Exclusion criteria were: history of active infection (eg. osteomyelitis, pulmonary tuberculosis, AIDS), chronic corticosteroids use, unstable angina or myocardial infarction in the last 3 months, advanced renal disease – defined as dialysis procedures, severe heart failure (classes 3 and 4), cirrhosis, alcohol or illicit drug use, dementia or difficult to full understand the studies procedures, current pregnancy or lactation, current cancer or any disease that might affect survival in the next 5 years.

At baseline, patients underwent an evaluation consisting of history and physical examination. Patients were considered as smokers or non-smokers. Ethnic definition was self-classified as white or non-white. Previous medical history was evaluated clinically. Cerebrovascular disease was established in the presence of a history of stroke and/or compatible findings (sequelae). The diagnosis of heart disease was based on a previous history of myocardial infarction, angina or heart failure and when available myocardial scintigraphy and coronary angiography. Body mass index (BMI) was calculated [weight (kg)/height^2^ (m)].

Blood pressure (BP) was measured twice at each visit in the sitting position after 10 minutes rest with OMRON Automatic Blood Pressure Monitor HEM- 720. Hypertension was defined as blood pressure levels ≥140/90 mmHg or use of anti-hypertensive drugs at screening visit. The protocol was approved by the Ethics Committee of Hospital de Clínicas de Porto Alegre and all patients provided written informed consent. This protocol was registered in Clinical Trials (ID 06260).

### Study design and interventions

This one-year, open-label, non-controlled, single-arm interventional study was conducted at a primary care unit located off-campus but in association with Hospital de Clínicas de Porto Alegre, a university hospital, in the metropolitan area of the city of Porto Alegre, Brazil. This unit is responsible for health care of approximately 40.000 individuals. Patients attending the primary care unit with diabetes and hypertension were invited to join the study.

The study comprised 3 stages: a run-in (3 months), drug intervention period (6 months) and stabilization period (2–3 months) and was conducted by an endocrinologist (LVV) and a generalist nurse (MFG). During the run-in period, patients were advised to maintain a healthy diet, to exercise and to take all the medications prescribed by their primary care physicians. Patients visited the primary care unit monthly and received orientation about diet, exercise and adherence to medication already in use. During the intervention period, participants visited the center at monthly intervals to check weight, BP, and glucose. The goal was to obtain systolic and diastolic BP ≤130/80 mmHg. If the mean systolic or diastolic BP values were higher than 130/80 mmHg, medications were administered in the following sequence: diuretic (hydrochlorothiazide); angiotensin converting enzyme (ACE) inhibitors (captopril or enalapril); β-adrenergic blocking agent (propranolol) and calcium channel blocking agent (amlopidine). These drugs are provided by the Brazilian public health care system. Medications were available at the primary care unit and patient was able to take it just after the consultation. The patients requiring more than four antihypertensive medications used hydralazine and/or clonidine (not available in the primary care unit). Medication was started with the lowest dose recommended by the manufacturer, and increased in increments until the maximum tolerated dose at monthly intervals guided by BP measurements. Another class of antihypertensive drugs was added after the maximum tolerated dose was reached. During the study period, patients received standard medical care in the primary care unit for intercurrences or other concomitant illness.

### Endpoints

Study endpoints were the change in systolic and diastolic BP after the intervention as well as the proportion of patients reaching and maintaining a BP ≤130/80 mmHg during the one-year study period.

### Laboratory methods

Fasting plasma glucose was measured by the glucose oxidase ultraviolet (UV) enzymatic method. Total cholesterol, HDL and triglycerides were measured by enzymatic methods. Low-density lipoprotein cholesterol (LDL) was calculated using the Friedewald equation. Serum creatinine was measured by a kinetic alkaline picrate method (Jaffe reaction) and converted to the standardized Jaffe Roche (CREA), traceable method, by linear regression equation (traceable Jaffe creatinine = − 0.236 + 1.061 × uncompensated Jaffe creatinine). Urinary albumin was measured in duplicate by immunoturbidimetric method (Microalb; Ames-Bayer, Tarrytown NY). Microalbuminuria was defined by a random spot urine sample higher than 17 mg/l [7,8]. All chemistry parameters were analyzed in a Modular P (Roche^®^ (Basel, Switzerland). The HbA1c test measurements (%) were performed by HPLC.

### Statistical analysis

Results are expressed as mean ± SD, median (P25-P75) or number of cases with the characteristic (%). Comparisons were performed by Student’s t test, Mann–Whitney U test or Chi-square test, as appropriate. Paired t-test was used to compare BP variation before and after intervention. P values <0.05 (two-sided) were considered to be statistically significant. SPSS 18.0 - Professional Statistics™ (SPSS Inc., Chicago, IL, USA) was used.

## Results

### Baseline characteristics

The original cohort comprised 116 diabetic patients of which 107 (92%) were diagnosed with hypertension. Mean hypertension duration was 10.7 ±10.4 years. The baseline characteristics of the hypertensive patients are shown in Table 1. Most patients were white (82.2%), 9 (8.4%) patients were smokers, and the mean BMI was 30.2 ± 5.9 kg/m^2^. At enrollment, diabetes treatment was diet alone in 11 patients, one oral agent in 46, two oral agents in 42, and three medications in 8; insulin was used in 23 (4 patients on insulin alone). Forty-four percent of the patients were on statins and mean LDL was 100.6 ± 28.4 mg/dl. Twenty-one patients (19.2%) had a previous cardiovascular event (stroke n = 4; ischemic heart disease n = 17; heart failure n = 3; lower limb amputation n = 1) and 25 patients (23.4%) were microalbuminuric. Mean systolic BP (SBP) and diastolic BP (DBP) were 145.3 ± 21.6 mmHg and 79.0 ± 11.4 mmHg, respectively, and BP lower than 130/80 mmHg was observed in 16 (15%) patients at the first visit. Hypertension medication previously prescribed by a primary care physician was as follows: no medication in 11 (10.3%) patients; one agent in 28 (26.2%); two agents in 46 (43%); three agents in 16 (15%) and four agents in 6 (5.5%) (69 patients on diuretics, 76 on ACE inhibitor, 36 on beta-blocker agent and 15 patients were using calcium channel blocking agents).

**Table 1:** Baseline clinical and laboratory characteristics of 107 hypertensive type 2 diabetic patients

**Baseline**	
N	107
Age (years)	62.6 ± 11.2
White ethnicity – n (%)	88 (82.2%)
Female sex– n (%)	69 (64.5%)
Diabetes duration (years)	8.3 ± 9.2
Primary care unit attendance (years)	2.3 ± 2.7
Previous cardiovascular event – n (%)	21 (19.2%)
Current Smoking – n (%)	9 (8.4%)
SBP (mmHg)	145.3 ± 21.6
DBP (mmHg)	79.0 ± 11.4
BMI (kg/m^2^)	30.2 ± 5.9
Using statin – n (%)	47 (44%)
Using aspirin– n (%)	66 (61.8%)
Microalbuminuria– n (%)	25 (23.4%)
Diabetes Treatment - n (%)	
Diet only	11 (10.3%)
One agent	46 (43%)
Two agents	42 (39.2)
Three agents	8 (7.5%)
Insulin use	23 (21.5%)
Hypertension Treatment	
No drugs	11 (10.3%)
One agent	28 (26.2%)
Two agents	46 (43%)
Three agents	16 (15%)
Four agents	6 (5.5%)
Diuretic	69
ACE inhibitor	76
β-blocker	36
Calcium channel blocking	15
Total cholesterol (mg/dl)	179.9 ± 39.7
HDL cholesterol (mg/dl)	48.0 ± 11.3
Triglycerides (mg/dl)	152 (107.3 -368.7)
LDL cholesterol (mg/dl)	100.6 ± 28.4
HbA1c (%)	7.3 ± 1.6

Data are mean ± SD, number of patients with the characteristic (%).

### Follow-up results

Of the 107 hypertensive patients that agreed to participate in the study, 29 (27%) were lost to follow-up and were not included in the final analysis [withdrawal of consent form (n = 3), lost to follow-up (n = 19), death (n = 2), stroke with important physical limitation (n = 1), and cancer (n = 4)]. Therefore, the results of the 78 patients (73%) that completed the trial are presented below. There was no difference between missing patients and those who completed the follow-up regarding age, sex, duration of hypertension and diabetes, and BP levels.

**Figure 1:**
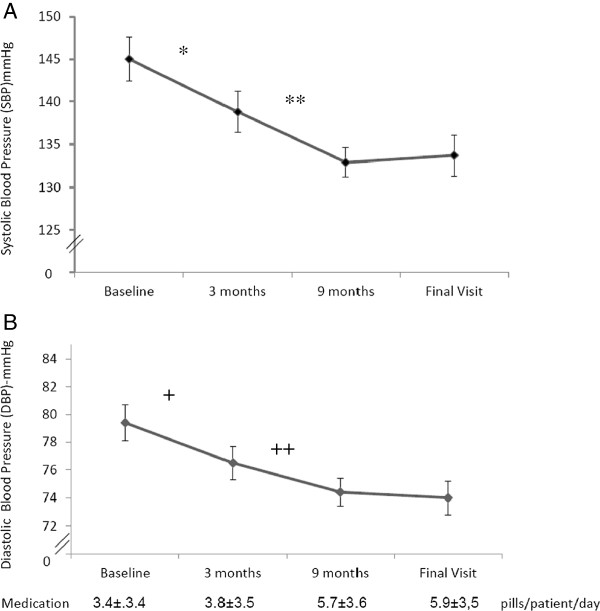
Blood Pressure decrease and Medication Tablets increase throughout the study: Panel A – Systolic Blood Pressure (mean ± SE); *Run-In: Systolic BP: 145.0 ± 22.8 vs. 138.8 ± 21.2 mmHg; p = 0.002; ** Intervention Systolic BP: 138.5 ± 21.21 to 132.6 ± 14.8 mmHg; P = 0.046; Panel B – Diastolic Blood Pressure (mean ± SE). +Run-In: Diastolic BP: 79.4 ± 11.5 vs. 76.5 ± 10.9; p = 0.026; ++ Intervention Diastolic BP: 76.4 ± 11.05 to 74.4 ± 8.6; P <0.01.

Changes in blood pressure are shown in Figure 1. From baseline to the end of the run-in period, there was a significant reduction in both systolic (145.0 ± 22.8 vs. 138.8 ± 21.2 mmHg; p = 0.002) and diastolic BP (79.4 ± 11.5 vs. 76.5 ±10.9; p = 0.026), yet no increase in the number of pills taken in this first part of the study was observed (3.4 ± 3.5 vs. 3.8 ± 3.5; p = 0.137). In the intervention period, the number of antihypertensive tablets increased (3.6 ± 3.5 vs. 5.9 ± 3.5 pills/patient; p <0.001), as the number of antihypertensive classes increased (1.8 ± 1.0 vs. 2.70 ± 1.2; p < 0.01). During this period, a further decline in SBP and DBP was observed and the overall drop of BP was 11 mm Hg for SBP (145.0 ± 22.8 vs. 133.7 ± 20.9 mmHg; p < 0.01) and 5 mmHg for DBP (78.7 ± 11.5 vs. 73.7 ± 10.5 mmHg; p = 0.001); the number of patients with BP values lower than 130/80 mmHg almost doubled [14 (18.7%) vs. 30 (38.5%) p = 0.008] from the first visit to the end of the study. During the stabilization period there was neither a decline in BP nor an increase in medication taken.

In order to identify baseline characteristics associated with better responses to the intervention, patients were stratified based on BP values at the end of the study. No differences regarding age, gender, and hypertension duration were seen between patients reaching BP goals, with the exception of a higher SBP at baseline in those with BP higher than 130 mmHg at the end of the study.

## Conclusion

In this cohort of type 2 diabetic and hypertensive patients, mean initial BP was 145/79 mmHg and only 15% of patients had ADA and JNC 7 target BP levels. During this one-year study, the number of patients who achieved the goal increased to 39%, with a mean drop of 11 mmHg in the SBP and 5 mmHg in DBP. The end-of-study BP was ~134/74 mmHg due to an important increase in the number of pills taken by these patients. It is worth noting that less than half the study participants ended the follow-up with a BP <130/80 mmHg.

Our baseline data is in agreement with previous results from a cross-sectional study conducted in Porto Alegre, Brazil, where 83% of the treated patients had BP levels higher than ADA goals [5]. A survey of NCHS examined hypertension management in diabetic patients and demonstrated that 66% of the diabetic patients visiting outpatient clinics had BP higher than the goals, with a mean of 139/78 mmHg. Interestingly, 71% of these patients were using antihypertensive agents, with nearly half involving prescription of 2 or more medications [9].

Therapeutic inertia is an important factor contributing to persistent elevated BP in these patients. In a cross-sectional study, researchers from Colorado found that more than 60% of type 2 diabetic patients did not achieve blood pressure goals, and actions to lower BP were taken in only 35% of the cases [10]. In our study, an aggressive BP lowering strategy, consisting of monthly evaluations and forced medication titration, doubled the number of patients with BP below the target. Two other strategies were tested in recently published trials to control BP in diabetic populations [11,12]. The addition of a pharmacist and a nurse to manage the patients cardiovascular risks in primary care resulted in BP reductions similar to those observed in the present study [11,12].

Another important factor that could contribute to the low number of patients on target is patient’s non-adherence to lifestyle modification strategies. The decrease in BP observed in the run-in period probably reflects an improvement in patient’s adherence to behavior modification reinforcements (lifestyle intervention) in the first 3 months of the study. There was no significant weight loss throughout the study despite orientation about diet and exercise at each appointment. Modifying diabetic patients diet is another important way to reduce BP. DASH diet applied to a diabetic population was able to reduce both systolic BP and diastolic blood pressure (−13.6 ± 3.5 mmHg; -9.5 ± 2.6 mmHg, respectively) [13].

Recently, BP targets in patients with diabetes and hypertension have been debated [14,15] because only one intervention study testing different BP goals was able to lower patients BP levels to ADA and JNC 7 recommendations [16]. In this scenario, a less strict blood pressure control (BP <140/80 mmHg or between 130-135 mmHg) as suggested by a recent meta-analysis [17] may be considered a more adequate target. Even when analyzed from this perspective (BP <140/80 mmHg) which matches the new recommendation from ADA 2013 [18], only 31% of our patients were initially well controlled and after the study intervention this number increased to 54%, leaving 46% of the patients at a higher risk for diabetic complications.

In conclusion, a BP algorithm applied to type 2 diabetic and hypertensive patients is able to lower BP, however more than half of the patients did not achieve the ADA and JNC 7 targets, demonstrating the complexity of BP control in this population. Revision of antihypertensive treatment strategies, perhaps employing a more aggressive life-style intensification strategy and/or including new classes of agents, is needed in order to guarantee an adequate BP control in patients with type 2 diabetes.

## Competing interests

The authors declare that they have no competing interests.

## Authors’ contributions

LVV researched data and wrote manuscript. MFG, EPPCR and JKB researched data. CBL, RF and JLG reviewed/edited the manuscript and contributed to discussion. All authors read and approved the final manuscript.
